# Energy Dispatch for CCHP System in Summer Based on Deep Reinforcement Learning

**DOI:** 10.3390/e25030544

**Published:** 2023-03-21

**Authors:** Wenzhong Gao, Yifan Lin

**Affiliations:** Merchant Marine College, Shanghai Maritime University, 1550 Haigang Avenue, Pudong District, Shanghai 201306, China

**Keywords:** deep reinforcement learning, DoubleDQN, CCHP system, energy dispatch, demand charge, uncertainties

## Abstract

Combined cooling, heating, and power (CCHP) system is an effective solution to solve energy and environmental problems. However, due to the demand-side load uncertainty, load-prediction error, environmental change, and demand charge, the energy dispatch optimization of the CCHP system is definitely a tough challenge. In view of this, this paper proposes a dispatch method based on the deep reinforcement learning (DRL) algorithm, DoubleDQN, to generate an optimal dispatch strategy for the CCHP system in the summer. By integrating DRL, this method does not require any prediction information, and can adapt to the load uncertainty. The simulation result shows that compared with strategies based on benchmark policies and DQN, the proposed dispatch strategy not only well preserves the thermal comfort, but also reduces the total intra-month cost by 0.13~31.32%, of which the demand charge is reduced by 2.19~46.57%. In addition, this method is proven to have the potential to be applied in the real world by testing under extended scenarios.

## 1. Introduction

The utilization of fossil fuels has caused environmental problems globally, such as air pollution and climate warming [1]. At the same time, energy consumption rises instead of falling with the rapid depletion of energy resources, of which about 40% is used for the production of cool, heat, and electricity [2]. Compared with the traditional energy supply system, the combined cooling, heating, and power (CCHP) system can promote the coordinated operation of the above three kinds of energy, which provides an effective way to solve environmental and energy problems with high energy efficiency. As a consequence, CCHP systems have been widely used in residential and office buildings, and hospitals in the past decade [3,4,5].

Although high energy efficiency has been verified in engineering applications, existing CCHP systems usually suffer from high operating costs, especially in summer. For example, Shanghai, a city in southern China, has a subtropical monsoon climate with outdoor temperatures up to 39 °C in summer, so a large amount of electricity is required to provide cooling to balance the demand-side user’s high cooling demand, which leads to the surge of CCHP system’s energy purchasing cost. Therefore, it is very necessary to optimize existing systems to achieve optimal economic operation in summer while meeting the demand-side energy load.

In the related literature, there are many studies focusing on optimizing the dispatch strategy of the CCHP system. Ref. [6] established a dispatch model of the CCHP system with the optimization goal of energy cost and carbon emission, and solved it by CPLEX solver. Ref. [7] established a two-stage dispatch model for the CCHP system based on quantity adjustment and proposed an iterative solution algorithm. Ref. [8] proposed an improved bee colony algorithm to solve the multi-objective dispatching model, so as to balance economic benefits and environmental friendliness. Ref. [9] built a load prediction model using neural networks, and proposed a feed-forward active operation optimization method considering load prediction for the CCHP system. Refs. [10,11,12] improved the traditional electric load following strategy and thermal load following strategy to improve the matching degree of energy output and demand. Ref. [13] used a genetic algorithm to optimize the dispatch strategy of the CCHP system to minimize the energy cost under different operating circumstances. In addition, because of human activities and outdoor weather conditions, there are dynamic uncertainties in the demand-side load, which brings a severe challenge to the energy dispatch of the CCHP system. To solve this problem, some studies tried to optimize the CCHP system dispatch strategy using model predictive control (MPC) [14,15,16] and robust optimization [17,18,19] separately, and the dispatch result showed that high-quality optimization depended on the accuracy of prediction.

Although the above studies have laid the solid foundation for the optimal energy dispatch of the CCHP system, the impact of demand charge on the operating cost is not considered. According to surveys, the so-called demand charge is already common among power industries in countries including China, the U.S, and Sweden, and is generally charged based on the electricity customers’ monthly peak power demand to the grid, that is, the peak electric power purchase (in kW) regardless of timing, rather than the cumulatively purchased electricity (in kWh) [20,21]. Additionally, some studies suggest that introducing demand charge into the utility rate has two potential benefits: (1) incentivizing smarter demand-side management; (2) guaranteeing the stability of the grid, and avoiding power accidents that may endanger public safety, such as large-scale blackouts [22,23]. The presence of demand charge further increases the difficulty of CCHP system energy dispatching. Therefore, in view of the above two factors, this paper will achieve the optimal economic operation of the CCHP system in summer under the rate structure including time-of-use electricity price and demand charge.

On the other hand, the optimization methods applied in all the above study works can be categorized as model-based methods. Although the model-based method well reflects the thermodynamic performance of the CCHP system and the internal mechanism of demand-side load variation, it relies on the expert experience of modeling or the prediction information of future uncertainty, which is difficult to adapt to dynamic changes of the actual environment. Once the operating status and structure of the CCHP system change with time, the model-based method needs to remake the dispatch strategy, which increases the computational burden and greatly reduces the decision-making efficiency. Besides, the model-based method often suffers from a low intelligence level and a long solution time, which cannot meet the requirements for real-time operation.

Therefore, in view of the above limitations, this paper introduces deep reinforcement learning (DRL) into the CCHP system energy dispatching problem. DRL is an emerging technique that has received extensive attention in recent years. With its strong perception and decision-making ability, DRL not only ensures real-time requirements, but also provides a model-free optimization approach to generate adaptive strategies without prior knowledge of the environment, avoiding drawbacks of the model-based method. In this sense, the DRL-based method has more advantages.

Studies have demonstrated that DRL can be used to solve complex high-dimensional control and optimization problems such as in robots [24], games [25], and traffic controls [26]. In the field of energy dispatching, DRL-based methods have also attracted broad attention. Ref. [27] proposed a microgrid energy management method based on a deep Q network to achieve higher economic benefits under stochastic conditions. Ref. [28] achieved the optimal control of the heating, ventilation, and air conditioning (HVAC) system using deep deterministic policy gradient (DDPG) to reduce energy costs and thermal discomfort. After improving the traditional DDPG algorithm, ref. [29] proposed a dynamic dispatch method for an integrated energy system based on improved DDPG, and verified the superiority of this method. Ref. [30] used DRL to optimize the electricity dispatch of an all-electric ferry, so as to achieve the dual-objective optimization of energy cost and load expected loss. Ref. [31] developed a microgrid energy management method using proximal policy optimization (PPO) to deal with the uncertainties of renewable energy. Ref. [32] developed a CHP system dispatch method using PPO, efficiently handling the wind-turbine failure without rewriting constraints. Although these study works have made significant contributions, they only considered a simple optimization objective of minimizing costs due to grid exchange and power generation, ignoring the impact of the demand charge mechanism and the control of peak exchanging power, which may result in an extra ultra-high cost. In addition, there is not only no benchmark policy designed to further verify the superiority and generalization of the proposed method, but also no comprehensive discussion of its potential application in real scenarios from multiple aspects.

Therefore, motivated by the above problems, this paper aims to develop a DRL-based energy dispatch method for the CCHP system to achieve optimal economic operation in summer. The innovation and main contributions are summarized as follows:

- We focus on the energy dispatch for the CCHP system in summer (EDCS) and formulate the EDSC problem as a Markov decision process (MDP), in which the load uncertainty, energy cost, demand charge, and energy balance are considered.
- DRL algorithm DoubleDQN is used to solve the formulated MDP and make dispatch strategies for the CCHP system. In contrast to previous study works, the proposed method directly makes decisions based on the current state, getting rid of the dependence on the accuracy of prediction information and model description.
- By comparing with the DQN-based method and benchmark policies, the advantages of the proposed method in computational efficiency, total intra-month cost saving, and peak power purchase control are verified.
- From the aspects of dealing with unseen physical environments, load fluctuation, and sudden unit failure, the potential of application in real scenarios is discussed.

The rest of the paper is organized as follows. The EDCS problem is mathematically formulated in Section 2; the proposed DRL-based method is introduced in Section 3; the case study is given in Section 4; finally, the conclusion is drawn in Section 5.

## 2. EDCS Problem Formulation

EDCS problem aims to efficiently manage the energy output of the CCHP system, to achieve optimal economic operation in summer. Therefore, this section first introduces a typical CCHP system, then establishes mathematical models of its key units, and finally designs the objective function and constraints of the EDCS problem.

### 2.1. System Description

#### 2.1.1. Structure of CCHP System

**Figure 1:**
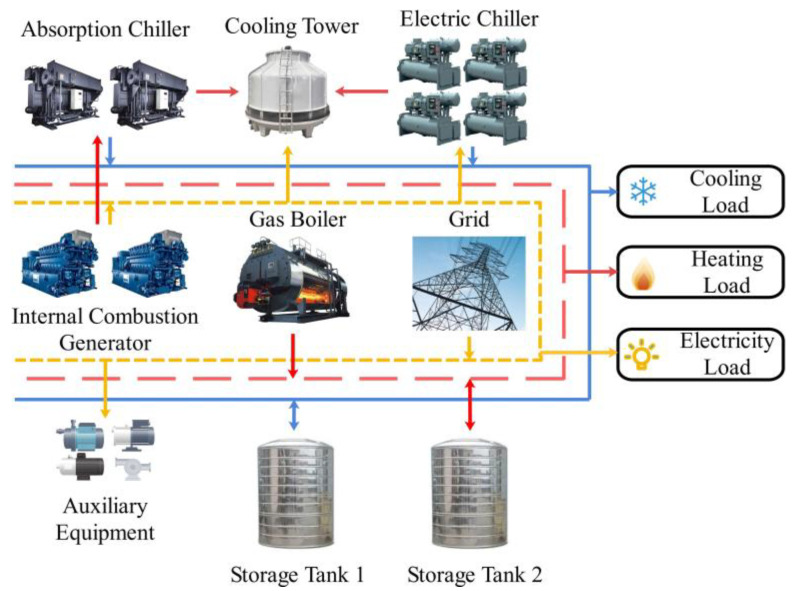
Structure and energy flow of the typical CCHP system.

Figure 1 shows a typical CCHP system, which consists of an internal combustion engine (ICE), absorption chiller (AC), electric chiller (EC), cooling tower (CT), gas boiler (GB), storage tank (ST) and auxiliary equipment (AE). The high-grade heat generated by the burning of natural gas is used to drive ICE to produce electricity, and the low-grade waste heat is used to produce cool through AC. If ICE fails to meet the electricity load, it will be supplemented by the grid. The cooling load is mainly satisfied by EC and AC, and the low-grade heat generated during the cooling process will be released through CT. The heating load is mainly satisfied by GB. Insufficient heating and cooling energy are supplied by ST, while the operation of this system is assisted by AE.

Additionally, the following assumptions are made for the typical CCHP system:

(1) Demand-side user is directly connected to the grid, and thus the electricity load only consists of ECs, AEs, and CT.

(2) ICEs and ECs run at rated power.

(3) ICEs and ACs run in a one-to-one matching mode, meaning the number of running ICEs is equal to the number of running ACs at each time step.

#### 2.1.2. Mathematical Model of Key Unit

Mathematical modeling is carried out for key units of the CCHP system under summer conditions, including ICE, AC, EC, ST, and AE:

(1) ICE

The ICE electric power output $P^{ICE}\left(t\right)\;$(kW) at time step *t* is defined according to the following equation:

(1) $$P^{ICE}\left(t\right)=\{\begin{array}{c} P_{rated}^{ICE},\;if\;\mathrm{ICE}\;is\;running\\ 0,otherwise \end{array}$$

where, $P_{rated}^{ICE}$ is the rated electric power generation of ICE (kW). Then, the ICE gas consumption $V^{ICE}\left(t\right)$ (m^3^/h) and the low-grade waste heat $Q^{waste}\;$(kW) can be calculated as follows:

(2) $$V^{ICE}\left(t\right)=\frac{P^{ICE}\left(t\right)}{\eta_{ICE}\cdot \varepsilon_{LHV}}$$

(3) $$Q^{waste}\left(t\right)=\left(\frac{1-\eta_{ICE}}{\eta_{ICE}}\right)\cdot P^{ICE}\left(t\right)$$

where, $\eta_{ICE}$ is the electric efficiency of ICE and $\varepsilon_{LHV}$ is the low calorific value of natural gas (kWh/m^3^).

(2) AC

The AC cooling output $Q^{AC}\left(t\right)$ (kW) at time step *t* is jointly decided by the absorbed waste heat $Q^{waste}\left(t\right)$ and the coefficient of performance $COP_{AC}$, that is:

(4) $$Q^{AC}\left(t\right)=Q^{waste}\left(t\right)\cdot COP_{AC}$$

(3) EC

The EC cooling output $Q^{EC}\left(t\right)\;$(kW) at time step *t* is defined according to the following equation:

(5) $$Q^{EC}\left(t\right)=\{\begin{array}{c} Q_{rated}^{EC},\;if\;\mathrm{EC}\;is\;running\\ 0,otherwise \end{array}$$

where, $Q_{rated}^{EC}$ is the rated cooling generation of EC (kW). Then, the EC electric power consumption $P_{e}^{EC}\left(t\right)$ (kW) can be calculated as the following equation:

(6) $$P_{e}^{EC}\left(t\right)=Q^{EC}\left(t\right)/COP_{EC}$$

where, $COP_{EC}$ is the coefficient of performance of EC.

(4) ST

The energy storage relationship of ST between adjacent time steps is defined as the following equation:

(7) $$E^{ST}\left(t\right)=E^{ST}\left(t-1\right)-Q^{ST}\left(t\right)\cdot \Delta t$$

where, Δt (h) is the time step interval, $E^{ST}\left(t\right)$ (kWh) and $Q^{ST}\left(t\right)$ (kW) are the remaining energy storage and the storing ($Q^{ST}\left(t\right)<0$) or releasing ($Q^{ST}\left(t\right)\geq 0$) power of ST at time step *t*, respectively.

(5) CT and AE

Since the running of CT and AE are closely related to the operating status of other energy supply units, the electricity consumption of CT and AE is allocated to ECs, ACs, ICEs, and ST for the convenience of calculation, where ICE and AC are allocated as a whole because of the matching mode, that is:

(8) $$P_{e}^{CT}\left(t\right)+P_{e}^{AE}\left(t\right)=P_{e,a}^{EC}\left(t\right)+P_{e,a}^{ICE\&AC}\left(t\right)+P_{e,a}^{ST}\left(t\right)$$

(9) $$P_{e,a}^{EC}\left(t\right)=\{\begin{array}{c} \alpha_{EC}\cdot {\left(n^{EC}\left(t\right)\right)}^{2}+\beta_{EC}\cdot n^{EC}\left(t\right)+\gamma_{EC},if\;n^{EC}\left(t\right)>0\\ 0,otherwise \end{array}$$

(10) $$P_{e,a}^{ICE\&AC}\left(t\right)=\{\begin{array}{c} \alpha_{ICE}\cdot {\left(n^{ICE}\left(t\right)\right)}^{2}+\beta_{ICE}\cdot n^{ICE}\left(t\right)+\gamma_{ICE},\;if\;n^{ICE}\left(t\right)>0\\ 0,\;otherwise \end{array}$$

(11) $$P_{e,a}^{ST}\left(t\right)=\{\begin{array}{c} \alpha_{ST}\cdot {\left(Q^{ST}\left(t\right)\right)}^{2}+\beta_{ST}\cdot \left(Q^{ST}\left(t\right)\right)+\gamma_{ST},\;\;\;if\;\;Q^{ST}\left(t\right)>0\\ 0,\;otherwise \end{array}$$

where, $P_{e}^{CT}\left(t\right)$ and $P_{e}^{AE}\left(t\right)$ are the electric power consumption (kW) of CT and AE, respectively; $P_{e,a}^{EC}\left(t\right)$, $P_{e,a}^{ICE\&AC}\left(t\right)$ and $P_{e,a}^{ST}\left(t\right)$ are the allocated electric power consumption (kW) of ECs, ACs, ICEs and ST, respectively; $n^{EC}\left(t\right)$ and $n^{ICE}\left(t\right)$ are the number of running ECs and running ICEs, respectively; α, β, γ are the electric power consumption coefficients.

### 2.2. Objective Function

Specifically, the objective of EDCS is to minimize the total intra-month cost $C_{total}^{CCHP}$ (RMB), which is composed of the total energy cost $C_{total}^{ECT}$ (RMB) and the total demand charge $C_{total}^{DC}$ (RMB), by optimally dispatching each unit of the CCHP system. So, the objective function can be defined as follows:

(12) $$min\;C_{total}^{CCHP}=min\;\left\{C_{total}^{ECT}+C_{total}^{DC}\right\}$$

$C_{total}^{ECT}$ can be calculated by:

(13) $$C_{total}^{ECT}=\sum_{t=1}^{T}c_{t}^{ECT}=\sum_{t=1}^{T}\left(\mu_{gas}\cdot \Delta t\cdot \sum_{i=1}^{n^{ICE}\left(t\right)}V_{i}^{ICE}\left(t\right)+\left(\{\begin{array}{c} \mu_{grid}\left(t\right)\cdot P^{grid}\left(t\right)\cdot \Delta t,\;if\;\;P_{grid}\left(t\right)>0\;\\ \mu_{sell}\cdot P^{grid}\left(t\right)\cdot \Delta t,\;otherwise\; \end{array}\right)\right)$$

where, *T* (h) is the total time steps in a dispatch period, $c_{t}^{ECT}$ (RMB) is the energy costs at time step *t*, $V_{i}^{ICE}\left(t\right)$ (m^3^/h) is the gas consumption of *i*th running ICE; $\mu_{gas}$ (RMB/m^3^), $\mu_{grid}\left(t\right)$ (RMB/kWh) and $\mu_{sell}$ (RMB/kWh) are the natural gas price, and the purchasing and selling electricity price, respectively.

$C_{total}^{DC}$ is obtained according to [33], and can be calculated by:

(14) $$C_{total}^{DC}=\mu_{demand}\cdot max_{1\leq t\leq T}\left\{\max\left(P^{grid}\left(t\right),0\right)\right\}$$

where, $max_{1\leq t\leq T}\left\{\max\left(P^{grid}\left(t\right),0\right)\right\}$ (kW) is the peak electric power purchase in a dispatch period, $\mu_{demand}$ (RMB/kW) is the unit price of the demand charge. Further, to allocate $C_{total}^{DC}$ to each time step [34], Equation (14) can be mathematically transformed into:

(15) $$C_{total}^{DCS}=\sum_{t=1}^{T}c_{t}^{DCS}=\mu_{demand}\cdot \sum_{t=1}^{T}\left(\frac{t}{T-1}\cdot P_{t}^{peak}-\frac{t-1}{T-1}\cdot P_{t-1}^{peak}\right)$$

(16) $$P_{t}^{peak}=max\left\{P_{t-1}^{peak},\max\left(0,P^{grid}\left(t\right)\;\right)\right\}$$

where, $c_{t}^{DCS}$ (RMB) is the demand charge at time step *t*; $P_{t}^{peak}$ is the peak electric power purchase (kW) in the last *t* time steps and defines $P_{0}^{peak}=0$.

Therefore, the EDCS problem objective function can be eventually expressed as:

(17) $$min\;C_{total}^{CCHP}=min\;\{\sum_{t=1}^{T}(c_{t}^{EC}+c_{t}^{DCS})\}$$

### 2.3. Constraints

#### 2.3.1. Energy balance Constraints

The energy balance constraints of the CCHP system under summer conditions can be listed as follows.

Cooling balance:

(18) $$\sum_{i=1}^{n^{EC}\left(t\right)}Q_{i}^{EC}\left(t\right)+\sum_{i=1}^{n^{ICE}\left(t\right)}Q_{i}^{AC}\left(t\right)+Q^{ST}\left(t\right)=Q_{d}\left(t\right)$$

where, $Q_{i}^{EC}\left(t\right)$ (kW) and $Q_{i}^{AC}\left(t\right)$ (kW) are the cooling output of *i*th running EC and *i*th running AC at time step *t*, respectively; $Q_{d}\left(t\right)$ is the cooling load (kW).

Electricity balance:

(19) $$\sum_{i=1}^{n^{ICE}\left(t\right)}P_{i}^{ICE}\left(t\right)+P^{grid}\left(t\right)=P_{d}\left(t\right)=\sum_{i=1}^{n^{EC}\left(t\right)}P_{e,i}^{EC}\left(t\right)+P_{e}^{CT}\left(t\right)+P_{e}^{AE}\left(t\right)$$

where, $P_{i}^{ICE}\left(t\right)$ (kW) and $P_{e,i}^{EC}\left(t\right)$ (kW) are the electric power output and consumption of *i*th running ICE and *i*th running EC, respectively; $P^{grid}\left(t\right)$ (kW) is the exchanging of electric power with the grid, $P^{grid}\left(t\right)>0$, if power is purchased, otherwise $P^{grid}\left(t\right)\leq 0$; $P_{d}\left(t\right)$ is the electricity load (kW).

#### 2.3.2. Operational Constraints

Besides energy balance constraints expressed in Equations (18) and (19), there are some operational constraints for energy supply units of the CCHP system.

ECs, ICEs, and ACs are constrained by the quantity. Hence,

(20) $$0\leq n^{EC}\left(t\right)\leq n_{max}^{EC}$$

(21) $$0\leq n^{ICE}\left(t\right)\leq n_{max}^{ICE}$$

(22) $$0\leq n^{AC}\left(t\right)\leq n_{max}^{AC}$$

where $n_{max}^{EC}$, $n_{max}^{ICE}$ and $n_{max}^{AC}$ are the maximum number of ECs, ICEs, and ACs, respectively.

ST is constrained by the capacity and the storing/releasing power. Hence,

(23) $$0\leq E^{ST}\left(t\right)\leq E_{rated}^{ST}$$

(24) $$\left|Q^{ST}\left(t\right)\right|\leq Q_{max}^{ST}$$

where, $Q_{max}^{ST}$ is the maximum storing/releasing the power of ST; $E_{rated}^{ST}$ is the rated capacity of ST and defines $E^{ST}\left(0\right)=0$.

## 3. DRL-Based EDSC Method

Since the EDCS objective function designed in Section 2.2 is to determine the output of energy supply units at each time step so as to minimize the total intra-month cost, the EDCS problem is essentially a sequential decision-making problem. In this section, we first convert the EDCS problem into a Markov decision process (MDP), in which the energy balance constraints and operational constraints of the CCHP system are considered. And the DRL algorithm is adopted to find the optimal strategy for this MDP.

### 3.1. Converting of EDCS Problem into MDP

An MDP is usually defined as a tuple 〈S,A,p,r〉, where S is the state space, A is the action space, p is the state transition probability, r: S×A→r is the reward function. The agent observes the current environment state s∈S and chooses an action a∈A(s), where A(s) represents the set of all admissible actions at state s [35].

**Figure 2:**
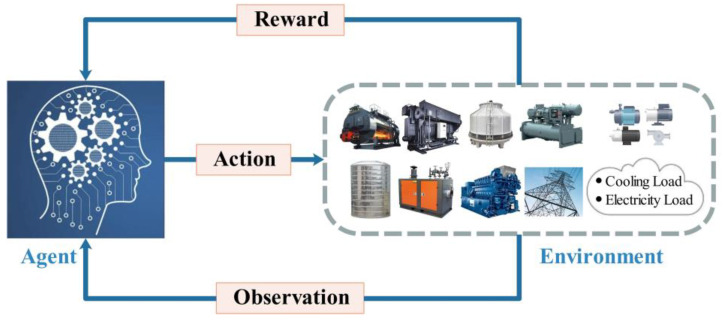
Interaction between the agent and the environment.

In this paper, the CCHP system is the environment where the agent is located, and the interaction between the agent and the environment is shown in Figure 2: at time step *t*, the agent observes the environment state $s_{t}$, and generates an action $a_{t}$ based on the policy π (policy is a mapping from state s to action a, that is, a=π(s)) to make dispatch strategy so as to determine the output of energy supply units.

Next, we convert the EDCS problem into an MDP, and the fundamental elements of which are defined as follows.

(1) State

At time step *t*, the environment state information for the agent includes physical time, remaining ST storage, peak electric power purchase obtained so far, purchasing electricity price, and cooling load. Among them, the cooling load is a state variable with uncertainty, which can’t be controlled by the agent. Thus, the state $s_{t}$ (5-dimension) is described as:

(25) $$s_{t}=\left[t,E^{ST}\left(t\right),P_{t-1}^{peak},\mu_{grid}\left(t\right),Q_{d}\left(t\right)\right]$$

(2) Action

The aim of the EDCS problem is to decide the electric power output ($\overset{n^{ICE}\left(t\right)}{\underset{i=1}{\sum }}P_{i}^{ICE}\left(t\right)+P^{grid}\left(t\right)$) and the cooling output ($\overset{n^{EC}\left(t\right)}{\underset{i=1}{\sum }}Q_{i}^{EC}\left(t\right)+\overset{n^{AC}\left(t\right)}{\underset{i=1}{\sum }}Q_{i}^{AC}\left(t\right)+Q^{ST}\left(t\right)$) of CCHP system. In fact, after $n^{ICE}\left(t\right)$ is decided, $\overset{n^{ICE}\left(t\right)}{\underset{i=1}{\sum }}P_{i}^{ICE}\left(t\right)$ and $\overset{n^{AC}\left(t\right)}{\underset{i=1}{\sum }}Q_{i}^{AC}\left(t\right)$ can be determined by Equations (1) and (4), respectively; after $n^{EC}\left(t\right)$ is decided, $\overset{n^{EC}\left(t\right)}{\underset{i=1}{\sum }}Q_{i}^{EC}\left(t\right)$ can be determined by Equation (5). Further, $Q^{ST}\left(t\right)$ and $P^{grid}\left(t\right)$ can be calculated by Equations (18) and (19), respectively. In other words, the output of other energy supply units can be obtained immediately after $n^{EC}\left(t\right)$ and $n^{ICE}\left(t\right)$ are jointly decided. Therefore, the agent’s action $a_{t}$ at time step t can be represented by $n^{EC}\left(t\right)$ and $n^{ICE}\left(t\right)$, that is:

(26) $$a_{t}=\left[n^{EC}\left(t\right),n^{ICE}\left(t\right)\right]$$

where, $a_{t}\in A$, and A is the set of all admissible actions that satisfy the EDCS constraints. According to Equations (20) and (21), $[0,\;n_{max}^{EC}$] and $[0,n_{max}^{ICE}$] are the range of $n^{EC}\left(t\right)$ and $n^{ICE}\left(t\right)$, respectively.

(3) Reward function

According to the EDCS objective function and constraints, the goal of the agent is to minimize the total cost which is composed of the energy cost and demand charge, while balancing the supply and demand of energy. In order to achieve this goal, the reward $r_{t}$ received by the agent consists of the above three parts, and can be defined as:

(27) $$r_{t}=-\lambda_{1}\cdot c_{EC}\left(t\right)+\left[-\lambda_{2}\cdot c_{DC}\left(t\right)-\theta \cdot \max\left(0,\;P_{t}^{peak}-\varepsilon_{peak}\right)\right]-\lambda_{3}\cdot \Delta Q_{d}\left(t\right)$$

where, $\lambda_{1}$, $\lambda_{2}$ and $\lambda_{3}$ are the weighting factors, $\Delta Q_{d}\left(t\right)$ is the cooling error (kW) (that is, the difference between supply and demand of cooling power); $\theta \cdot \max\left(0,\;P_{t}^{peak}-\varepsilon_{peak}\right)$ is the extra penalty obtained when the peak power purchase $P_{t}^{peak}$ is larger than the threshold $\varepsilon_{peak}$. The setting of $r_{t}$ transfers the total intra-month cost minimization problem to the reward maximization form of the MDP.

From the viewpoint of MDP, the quality of chosen action a at the given environment state s can be evaluated by the state-action value function $Q_{\pi }\left(s,a\right)$:

(28) $$Q_{\pi }\left(s,a\right)=E_{\pi }\left[\sum_{k=0}^{T}\tau^{k}\cdot r_{t+k}|s_{t}=s,a_{t}=a\right]$$

where, τϵ[0,1] is the discount factor used to balance the future reward and current received reward [36], $E_{\pi }\left[\cdot \right]$ is the reward expectation under the policy π. The aim of the agent is to find an optimal policy $\pi^{*}$, so as to maximize the function $Q_{\pi }\left(s,a\right)$, that is, $\pi^{*}=argmax_{a\in A}Q_{\pi }\left(s,a\right)$.

On the other hand, the above MDP doesn’t define the state transition probability p. With a full knowledge of p, the model-based method can solve $E_{\pi }\left[\overset{T}{\underset{k=0}{\sum }}\tau^{k}\cdot r_{t+k}|s_{t}=s,a_{t}=a\right]$ (that is, the $Q_{\pi }$ value of action a) through fully observing the environment [37]. However, due to the influence of dynamic uncertainty factors such as human activity, environmental weather, and unit failure, the establishment of p model becomes extremely difficult, which makes the model-based method, not a suitable solution. 

Therefore, in this paper, the model-free DRL-based method is used to solve the EDCS problem under the MDP framework. By interacting with the environment, the DRL-based method can incrementally improve its decision strategy without any information on state transition probability p.

### 3.2. DRL Solution

#### 3.2.1. A Brief Review of DRL

Reinforcement learning (RL) is a paradigm of machine learning. With the assistance of the Q table, the RL algorithm can iteratively update the state-action value function based on the reward function defined in MDP [38]:

(29) $$Q\left(s_{t},a_{t}\right)\;\leftarrow \;Q\left(s_{t},a_{t}\right)+\psi \cdot \left[r\left(s_{t},a_{t}\right)+\tau \cdot max_{a\in A}Q\left(s_{t+1},a_{t+1}\right)-Q\left(s_{t},a_{t}\right)\right]$$

where, ψϵ[0,1] is the learning factor.

DRL is the combination of deep neural network (DNN) and RL [39], the essence of which is to approximate Q(s,a) through the nonlinear function. When encountering problems with large state space, DRL utilizes DNN as the regression tool, which solves the potential dimension-explosion problem of the RL algorithm caused by the establishment of a huge Q table [40].

Additionally, DRL can be generally divided into the value-based DRL algorithm for discrete action space and the policy-based DRL algorithm for continuous action space [37,41]. Since the action space defined in Equation (25) is discrete, the value-based DRL algorithm is adopted to generate a dispatch strategy for the CCHP system.

#### 3.2.2. Basic Principles of Value-Based DRL Algorithm

**Figure 3:**
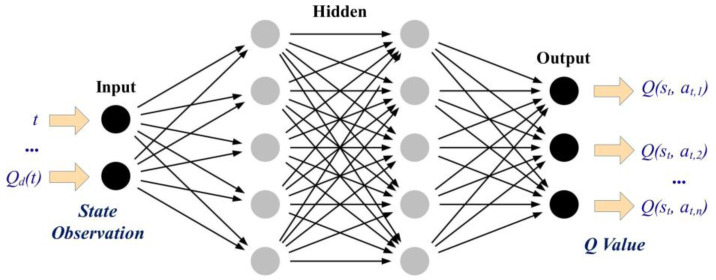
General DNN structure for the value-based DRL algorithm.

A general DNN structure for the value-based DRL algorithm is shown in Figure 3. Specifically, DNN is used to evaluate the Q value of each potential action corresponding to the state s, and the agent will select the action with the highest Q value.

A deep Q network (DQN) is a representative value-based DRL algorithm [42], which has two DNNs named Q network and the target network. The training objective of DQN is to minimize the loss function L(ω):

(30) $$\{\begin{array}{c} L\left(\omega \right)=E\left[y_{t}-Q\left(s_{t},a_{t};\omega \right)\right){]}^{2}\\ \;y_{t}=r_{t}+\tau \cdot max_{a}Q\left(s_{t+1},a;\omega^{\prime }\right) \end{array}$$

where, $y_{t}$ is the target Q value, $y_{t}-Q\left(s_{t},a_{t};\omega \right)$ is the time-difference error; ω and $\omega^{\prime }$ are weight parameters of the Q network and target network, respectively.

However, as shown in Equation (30), Q values used for action evaluation and selection in DQN are both outputs by the target network, which tends to cause overvaluation. In order to solve this problem, a greedy-policy-based double deep Q network (DoubleDQN) algorithm is proposed to decouple the evaluation and selection [43]. DoubleDQN evaluates the Q value using the Q network and selects the action to take using the target network. The target Q value is then:

(31) $$y_{t}=r_{t}+\tau \cdot Q\left(s_{t+1},argmax_{a}Q\left(s_{t+1},a;\omega \right);\omega^{\prime }\right)$$

and the gradient $\nabla_{\omega }L\left(\omega \right)$ provides the direction for DNN parameters updating, that is:

(32) $$\{\begin{array}{c} \nabla_{\omega }L\left(\omega \right)=E\left[2\cdot \left(y_{t}-Q\left(s_{t},a_{t};\omega \right)\right)\cdot \nabla_{\omega }Q\left(s_{t},a_{t};\omega \right)\right]\\ \omega \leftarrow \omega -\psi \cdot \nabla_{\omega }L\left(\omega \right) \end{array}$$

where, weights ω is updated every step and copied to weights $\omega^{\prime }$ every fixed number of steps. Additionally, a mechanism called experience replay is integrated into DoubleDQN [44]. In this mechanism, the agent stores the experience $e_{t}=\left(s_{t},a_{t},r_{t},s_{t+1}\right)$ at each time step, and randomly extracts a batch of experience samples for the off-line training.

#### 3.2.3. Realizing EDCS with DoubleDQN

**Figure 4:**
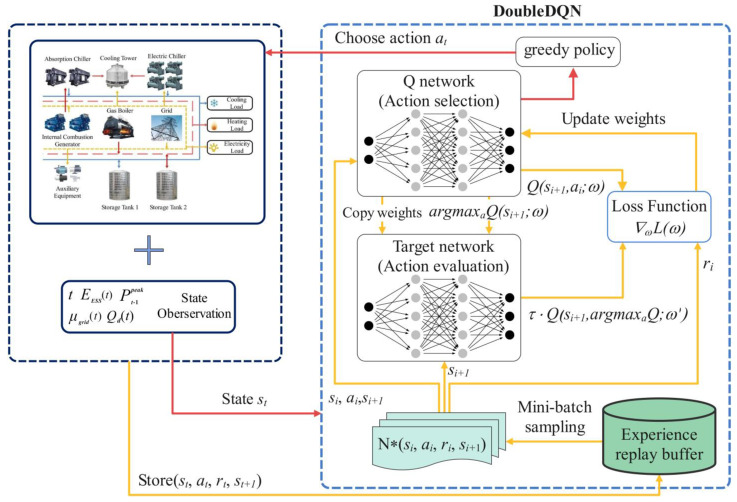
Framework of the proposed DoubleDQN-based EDCS method.

The framework of the DoubleDQN-based EDCS method is shown in Figure 4. For the DoubleDQN network, the input is a 5-dimensional vector $s_{t}=\left[t,E^{ST}\left(t\right),P_{t-1}^{peak},\mu_{grid}\left(t\right),Q_{d}\left(t\right)\right]$, and the outputs are the Q values of all potential actions.

We use historical data of the CCHP system as environment states to train the DoubleDQN algorithm offline. Its input includes physical time, ST storage, peak electric power purchase, electricity price, and cooling load. After the offline training process shown in Algorithm 1, the parameters of DoubleDQN will be fixed and used for the online decision-making of the CCHP system.
**Algorithm 1** Offline-training process of the DoubleDQN algorithm1:Initialize parameters of Q network (ω) and target network ($\omega^{\prime }$).2:**for** episode = 1 to M **do**:3:    Initialize $s_{1}=\left[1,E_{ESS}\left(1\right),P_{0}^{peak},\mu_{grid}\left(1\right),Q_{d}\left(1\right)\right]$.4:    **for**
*t* = 1 to *T*
**do**:5:          Select action $a_{t}$ at given $s_{t}$ using the greedy policy.6:          Execute $a_{t}$ in the CCHP environment and transit to the next state $s_{t+1}$.7:          Get reward $r_{t}$.8:          Store the experience ($s_{t},a_{t},r_{t},s_{t+1}$) in the experience replay buffer.9:          Extract a mini-batch of experience ($s_{t}^{i},a_{t}^{i},r_{t}^{i},s_{t+1}^{i}$) with the size *N* from the experience replay buffer.10:          Calculate the loss function: $L\left(\omega \right)=E\left[r_{t}+\tau \cdot Q\left(s_{t+1},argmax_{a}Q\left(s_{t+1},a;\omega \right);\omega^{\prime }\right)-Q\left(s_{t},a_{t};\omega \right)\right){]}^{2}$. 11:          Update the weights of the Q network: $\omega =\omega -\psi \cdot \nabla_{\omega }L\left(\omega \right)$.12:          Copy the weights ω into the target network every fixed number of time steps: $\omega^{\prime }=\omega$
13:    **end for**
14:**end for**

The decision-making procedure of the proposed DoubleDQN-based EDCS method can be found in Algorithm 2. When the dispatch begins, the weights ω of the Q network trained by Algorithm 1 are loaded. At each time step *t*, the agent selects an action $a_{t}$ based on the current CCHP state $s_{t}$. Next, the action is executed by energy supply units, and the CCHP environment transits to the next state $s_{t+1}$. Meanwhile, the agent receives the reward $r_{t}$ and observes $s_{t+1}$ as the current state. This procedure repeats until the end of the dispatch period. From the procedure, it can be found that the proposed method requires no prediction information, realizing a direct mapping from the real-time state observation to the CCHP system energy dispatching.
**Algorithm 2** Decision-making procedure of the proposed method**Input**:Environment state observation $s_{t}$ of time step *t*.**Output**:Dispatch decision $a_{t}$ for energy supply units.1:Load the weights ω of the Q network trained by Algorithm 1.2:**for** time step = 1 to *T* **do**:3:    Select action $a_{t}=\pi \left(s_{t};\omega \right)$.4:    Execute $a_{t}$ in the CCHP environment and transit to the next state $s_{t+1}$.5:    Get reward $r_{t}$.6:**end for**

## 4. Case Study

In this section, the effectiveness and superiority of the proposed method are verified by comparison with the designed DQN-based method and benchmark policies. In addition, the proposed method is further tested under extended scenarios.

### 4.1. Simulation Setup

In order to evaluate the proposed DoubleDQN-based EDCS method, a CCHP system located in EXPO Site (Shanghai, China) is taken as a subject for the case study, the structure of which is shown in Figure 1. The system provides cooling for 28 office buildings on the site, and their working hours of them are from 6:00 to 18:00 on weekdays. This paper uses the historical cooling load data of these buildings from 2018 to 2020 for the proposed method of training and testing. The cooling load data from May to July is used to train, and the cooling load data from August is used to test. The length of the dispatch period is 720 h (that is, a full month). In addition, considering the buildings’ working hours, 19:00 on the previous month’s last day and 18:00 on the current month’s last day are regarded as the start and end indexes of the dispatch period, respectively.

The CCHP system parameters are provided in Table 1. Additionally, the demand charge unit price $\mu_{demand}$, the natural gas price $\mu_{gas}$ and the selling electricity price $\mu_{sell}$ are 42 RMB/kW, 2.57 RMB/m^3^, and 0.568 RMB/kWh, respectively. The purchasing electricity price $\mu_{grid}$ is the time-of-use price: the valley tariff is 0.232 RMB/kWh (22:00~5:00), the peak tariff is 1.062 RMB/kWh (8:00~10:00, 13:00~14:00 and 18:00~20:00), and the flat tariff is 0.716 RMB/kWh at all other times.

The hyperparameters of DoubleDQN are shown in Table 2. Meanwhile, two common DRL algorithms DQN and dueling deep Q network (DuelingDQN) are introduced for use in subsequent subsections. Their hyperparameters are consistent with those of DoubleDQN.

The proposed algorithm is implemented using the deep learning framework PyTorch 1.8.0, and the simulation experiments are carried out on a computer equipped with AMD Ryzen7 5700U CPU and 16 G RAM.

### 4.2. Off-Line Training Process

**Figure 5:**
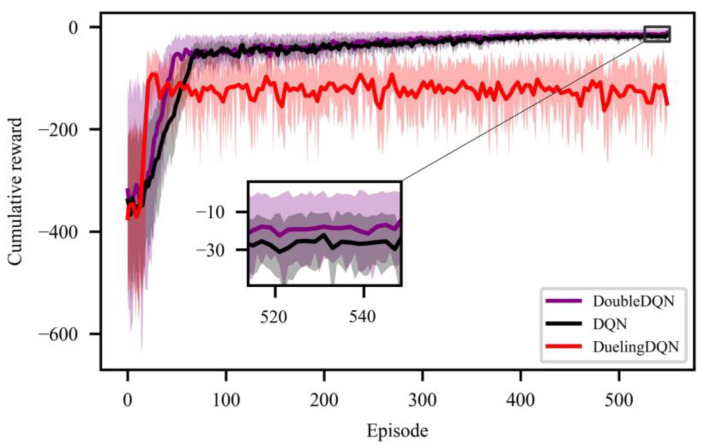
Cumulative reward performance of DoubleDQN, DQN, and DuelingDQN.

Figure 5 shows the cumulative reward performance of DoubleDQN, DQN, and DuelingDQN during the offline training process, where the full line represents the average. In the beginning, since the DRL agent is unfamiliar with the environment, the selected action results in a large variation in cumulative reward. As the training process continues, the agent begins to optimize the strategy to accumulate greater rewards. After about 32 episodes, DuelingDQN starts to converge, which is slightly prior to DoubleDQN (45 episodes) and DQN (68 episodes). However, DoubleDQN eventually obtains the greatest cumulative reward at convergence, which is far higher than that of DuelingDQN. This shows DoubleDQN’s superior learning performance in exploring the optimal strategy. Additionally, due to DuelingDQN’s poor cumulative reward performance, it is not considered in subsequent sections.

### 4.3. Dispatch Result Evaluation

Well-trained DRL agents from both the DoubleDQN and DQN are run during the test month (August 2020) to evaluate their dispatch results. Additionally, two benchmark policies are designed for comparison. The benchmark policies are described as follows: (a) Rule-based policy: during the valley electricity price period, ECs are given the priority to providing cooling, followed by ST and ACs, and the priority is completely reversed at all other times; (b) Shortsighted policy (based on DRL): the reward function defined in Equation (27) only consists of the energy cost and cooling error, and DRL agents are constructed using DQN and DoubleDQN, respectively.

The dispatch results and computational time of each method above are shown in Table 3, where the cooling error is expressed by the ratio of the total error to the total load, and the shortsighted policy is identified by the letter “a”.

As observed from Table 3, the three DRL methods have a significant advantage in online running time. Compared with the rule-based policy, the average time for DoubleDQN, DoubleDQN-a, DQN, and DQN-a to generate a set of dispatch decisions is reduced by 92.89%, 92.79%, 93.04%, and 92.45%, respectively. That’s because the rule-based policy has to take a few seconds at each time step to make a series of logical judgments based on different environment information, so as to output the dispatch strategy. On the contrary, the DRL-based methods can make decisions in less than 4.25 ms by using the DNN that is well trained during the off-line training phase, which is far less than the dispatch interval of the rule-based policy. So, they can meet the requirements for real-time operation better.

It can be also found that the rule-based policy outperforms all DRL-based methods in controlling the cooling error. However, since it can only rigidly make the dispatch strategy according to the electricity price signal, the highest total cost is obtained as a consequence. On the other hand, compared to the rule-based policy, DoubleDQN-a saves 34.69% in energy cost while DoubleDQN saves only 32.53%, and a similar difference holds comparing DQN-a to DQN. It suggests that the shortsighted policy can better save energy costs and is more suitable for scenes without demand charges. However, DoubleDQN shows a greater advantage in the demand charge, which is 2.19~46.57% lower than other methods, thus obtaining the lowest total intra-month cost. Additionally, DoubleDQN also controls the cooling error at a relatively low level, indicating the demand-side user’s comfort zone is well preserved.

**Figure 6:**
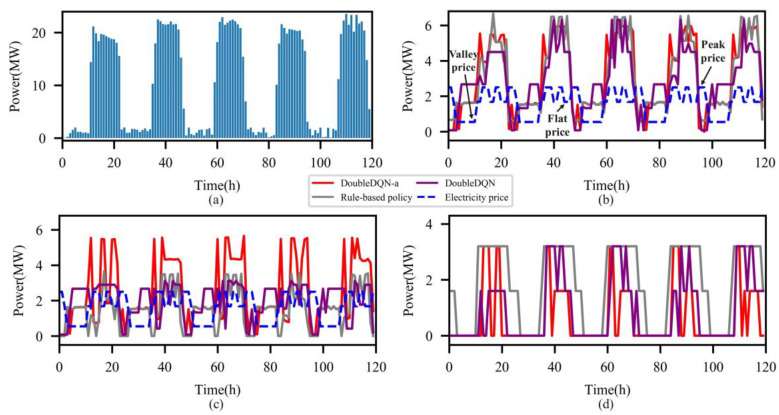
Electricity dispatch strategies of three methods for a typical week: (**a**) cooling load (**b**) electricity load (**c**) electric power purchase (**d**) electric power generation.

To explore DoubleDQN’s advantage in the demand charge, we compare the electricity dispatch strategies of DoubleDQN, DoubleDQN-a, and rule-based policy for a typical week of the test month, as shown in Figure 6. As observed, under DoubleDQN-a’s dispatch strategy, the grid mainly supplies the electricity load, and the electric power purchase in the flat electricity price period is far higher than other methods, especially reaching a peak of 5739 kW in 70 h. However, under DoubleDQN’s dispatch strategy, the peak electric power purchase is reduced from 3578 kW (rule-based policy) and 5739 kW (DoubleDQN-a) to 3112 kW by increasing ICEs’ total electric power output, which greatly reduces the demand charge and flattens the electric power purchase curve. Additionally, part of the electricity load under this strategy is shifted to the nighttime when both the cooling load and electricity price are relatively low, which saves energy costs and maintains electricity stability. Therefore, through the above comparison, it can be easily found that DoubleDQN exhibits better capabilities of demand response and peak demand management.

**Figure 7:**
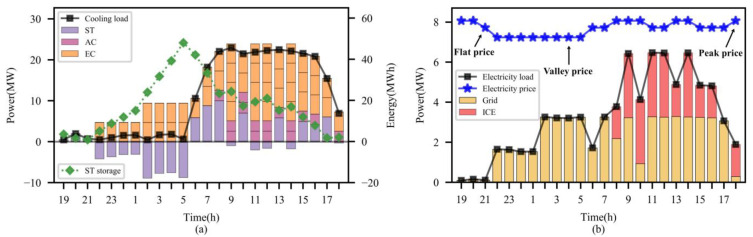
Dispatch strategy of DoubleDQN for a typical day: (**a**) cooling load balance (**b**) electricity load balance.

Figure 7 further illustrated the dispatch strategy of DoubleDQN for a typical day of the test month. It can be observed that the supply and demand of electric and cooling power are balanced throughout the day. As the electricity purchasing price is in the valley period (19:00~5:00), ECs consume the electric power purchased from the grid for providing cooling, and the redundant energy is stored in ST to release when requiring more cooling (6:00~17:00). When the cooling load and electricity price are both relatively high (9:00~16:00), ECs maintain the high total cooling output, while ICEs consume the natural gas for electric power generation to curb the peak electric power purchase, and the waste heat is used to produce cool through ACs in the corresponding period. This shows that DoubleDQN can flexibly manage the output of multiple units according to the environment information, so as to achieve the optimal energy dispatch.

### 4.4. Extending the Proposed Method to Different Scenarios

In this subsection, different scenarios are designed to further test the proposed DoubleDQN-based method, and the test month is still applied. The scenarios are:

Scenario C_1_: The proposed method is tested in new CCHP system models generated with different system parameters to test its generalization.

Scenario C_2_: The cooling load at each time step *t* randomly fluctuates in the range of [1−α%,1+α%] to test the proposed method’s robustness to the uncertain load, where α∈{5, 10, 15, 20}.

Scenario C_3_: Three typical unit failures are designed to test the proposed method’s effectiveness in handling sudden unit failure. Those three-unit failures occur randomly at each time step, and the probability distribution of which is shown in Table 4, where the value pair (A, B) represents the maximum runnable number of ECs and ICEs at time step *t*, respectively.

#### 4.4.1. Scenario C_1_

**Figure 8:**
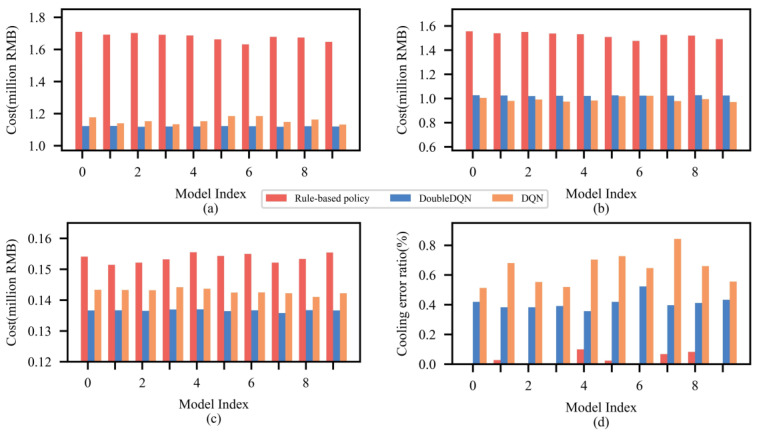
Comparison of dispatch results from DoubleDQN, DQN and rule-based policy: (**a**) total cost (**b**) energy cost (**c**) demand charge (**d**) cooling error ratio.

Ten new CCHP system models are generated with different system parameters, and the variation of which follows a normal distribution N(ζ, 0.1ζ), where ζ is the raw parameter. The dispatch results for these 10 models under DoubleDQN, DQN and rule-based policy are compared in Figure 8. It can be observed that similar to the results in Table 3, the rule-based policy obtains the lowest cooling error, while the highest energy cost and demand charge are obtained at the same time; DQN obtains the lowest energy cost and highest cooling error. In contrast, DoubleDQN can properly balance the above three objectives, and thus stably bringing the lowest total cost and relatively lower cooling error to all CCHP system models. Therefore, it can be easily concluded that DoubleDQN can adapt to unseen physical environments after being trained offline in a fixed environment.

#### 4.4.2. Scenario C_2_

For each load fluctuation degree α, 150 experiments are carried out respectively, and the MPC-based method, which is widely used to solve uncertainty problems, is introduced for the comparison. MPC-based method: (a) making dispatch decisions based on the rolling load-prediction, and the selected rolling horizon is set as 4 h; (b) the load-prediction error follows a normal distribution N(0, 0.2σ), where σ is the actual load value.

**Figure 9:**
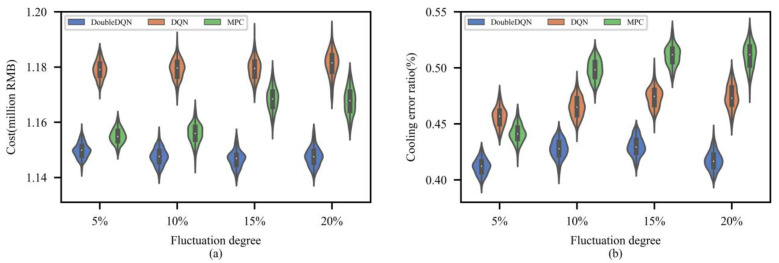
Comparison of dispatch results from DoubleDQN, DQN, and MPC: (**a**) total cost (**b**) cooling error ratio.

As shown in Figure 9, the dispatch results of DoubleDQN, DQN and MPC in a total of 600 experiments are plotted and compared by violin plot. It can be observed that with the increase of load fluctuation degree α, the three methods’ distributions of total cost and cooling error become more divergent. However, compared with DQN and MPC, the results of DoubleDQN show no significant discretization and achieve the lowest mean instead. Especially when the load fluctuation degree α is 5%, the superiority of DoubleDQN is more prominent. This shows that, unlike the MPC-based method, which relies on load-forecast accuracy, DoubleDQN efficiently deals with the uncertain load by directly making dispatch decisions based on real-time observations, and thus provides the dispatch strategy with higher stability and economic benefits. So, the proposed method can be relatively easier applied in real-world scenarios, especially when the prediction information is noisy or even missing.

#### 4.4.3. Scenario C_3_

For the unit failures defined in Table 4, the corresponding DRL agents are constructed using DoubleDQN and DQN respectively to be called on-demand during the operation period of CCHP system. The dispatch results of DoubleDQN, DQN and rule-based policy in 150 experiments are compared in Table 5.

It can be seen from the table that the average total cost of DoubleDQN is 2.72% and 31.51% lower than that of DQN and the rule-based policy, respectively. Meanwhile, DoubleDQN also achieves better performance than these methods in terms of minimum and maximum, maintaining relatively high economic benefits. On the other hand, compared with DQN, DoubleDQN has obtained a performance closer to that of the rule-based policy in controlling cooling error, well preserving the comfort zone for the demand-side user.

**Figure 10:**
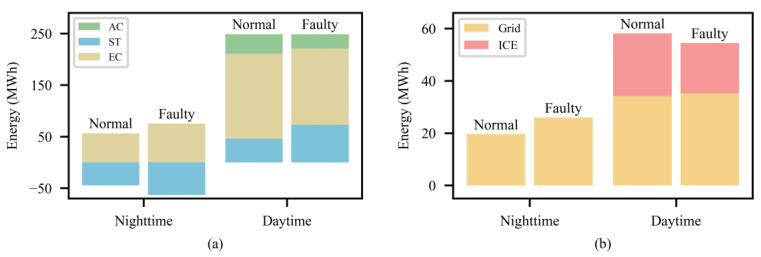
Dispatch strategies of DoubleDQN both under normal and faulty conditions: (**a**) normal condition (**b**) faulty condition.

Figure 10 further compares the dispatch strategies of DoubleDQN for a typical day both under normal and faulty conditions, i.e., sudden unit failures. Under the faulty condition, DoubleDQN increases the energy storage of ST by 42% by increasing the cool production of ECs during the nighttime. Therefore, the higher storage allows ST to release more cooling energy than the normal condition, so as to reduce the cool production of ECs and ACs by 11% and 19% during the daytime, respectively. On the other hand, the electricity load shows a trend of shifting from the daytime to the nighttime accordingly, which reduces the purchasing electricity cost and the daytime ICE electricity generation. Therefore, it can be easily concluded that by rationally planning the energy storage and release of ST, DoubleDQN reduces the CCHP system’s dependence degree of both ECs and ICEs while balancing the supply and demand, so as to efficiently handle unpredictable unit failures during the operation period, which can meet the requirements for practical application.

## 5. Conclusions

This paper focuses on the summer energy dispatching problem of the CCHP system. Aiming at minimizing the total intra-month cost and balancing the supply and demand of energy, a model-free DoubleDQN-based method is proposed to generate an optimal dispatch strategy. Different from the traditional method, this method makes dispatch decisions directly based on the real-time observed electricity price and cooling load, avoiding the suboptimal dispatching problem caused by prediction error. Through the simulation results, the following conclusions can be drawn:

(1) Compared with DRL algorithms DQN and DuelingDQN, DoubleDQN shows better learning performance during off-line training and obtains the greatest cumulative reward at convergence.

(2) The proposed method shows good demand response and peak shift ability. By restraining the peak electric power purchase of the CCHP system to below 3112 kW, the total intra-month cost is further reduced by 0.13~31.32% compared with the designed DRL methods and the rule-based policy through greater demand charge advantage. In addition, the method also considers the decision speed and thermal comfort, which not only meets the requirements of real-time operation but also well preserves the comfort zone for the demand-side user.

(3) In dealing with unknown system parameters, load uncertainty and sudden unit failure, the proposed method provides the dispatch strategy with higher stability and economic benefits for the CCHP system, showing strong generalization and potential for application in real scenarios.

On the other hand, the future study work will focus on two directions: one is to focus on the improvement of the traditional DRL algorithm to reduce the time and computing resources in DRL training; the other is to include environmental pollution into optimization indicators.

## Figures and Tables

**Table 1:** CCHP system parameters.

Parameter	Value	Parameter	Value
$\eta_{ICE}$	0.46	$\alpha_{EC},\beta_{EC},\gamma_{EC}$	0.013, 474.293, 1.615
$n_{max}^{ICE},n_{max}^{EC},n_{max}^{AC}$	2, 4, 2	$\alpha_{ICE},\beta_{ICE},\gamma_{ICE}$	−1.021, 1312.225, 0.005
$P_{rated}^{ICE},Q_{rated}^{EC}$ (kW)	1600, 4700	$\alpha_{ST},\beta_{ST},\gamma_{ST}$	0.005, 0.062, 2.970
$Q_{max}^{ST}$ (kWh)	10,000	$COP_{EC}$	5.2
$E_{rated}^{ST}$ (kWh)	70,000	$COP_{AC}$	1.3

**Table 2:** Hyperparameters of DoubleDQN.

Description	Training Value	Description	Training Value
Size of input	5	Mini-batch size	128
No. of hidden layers	3	Discount factor	0.925
Size of each hidden layer	128, 512, 128	Learning rate	0.0005
Size of output	2	Weights of reward	$\lambda_{1}$: 5 × 10^−5^, $\lambda_{2}$: 6 × 10^−4^, θ: 0.002, $\varepsilon_{peak}$: 3300
Activation function for each hidden layer	ReLU	Optimizer	Adam

**Table 3:** Dispatch results and computational time of each method.

Method	Total Cost (RMB)	Energy Cost (RMB)	Demand Charge (RMB)	Rate of Cooling Error (%)	Total Online Running Time (s)	Total Offline Training Time (s)
Rule-based policy	1,678,249	1,524,251	153,998	0	20.27	-
DoubleDQN	1,152,830	1,013,176	139,654	0.312	1.44	973
DoubleDQN-a	1,256,845	995,455	261,390	0.608	1.46	995
DQN	1,154,421	1,011,638	142,783	0.550	1.41	1204
DQN-a	1,263,219	993,489	262,730	0.652	1.53	1013

**Table 4:** Probability distribution of unit failures.

Unit Failure (A, B)	Probability (%)
(4, 1)	35
(3, 2)	15
(3, 1)	5

**Table 5:** Dispatch results of each method.

Method	Total Cost (RMB)			Ratio of Cooling Error (%)		
	Min	Mean	Max	Min	Mean	Max
DoubleDQN	**1,148,908**	**1,152,980**	**1,158,866**	**0.292**	**0.412**	**0.531**
DQN	1,168,379	1,185,324	1,189,573	0.358	0.659	1.031
Rule-based policy	1,644,612	1,683,547	1,691,356	0.014	0.305	0.804

## Data Availability

The data presented in this study are available on request from the corresponding author. The data are not publicly available due to privacy.

## Nomenclature

AC	absorption chiller	$P_{t}^{peak}$	peak electric power purchase in last *t* time steps, kW
AE	auxiliary equipment		
CCHP	combined cooling, heating and power	$Q_{d}$	cooling load, kW
COP	coefficient of performance	$Q_{i}^{AC}$	cooling output of *i*th running AC, kW
CT	cooling tower	$Q_{i}^{EC}$	cooling output of *i*th running EC, kW
DC	demand charge	$Q_{rated}^{EC}$	rated cooling generation of EC, kW
DRL	Deep reinforcement learning	$Q^{ST}$	storing/releasing power of ST, kW
EC	electric chiller	$Q^{waste}$	low-grade waste heat, kW
ECT	energy cost	$Q_{\pi }\left(s,a\right)$	state-action value function
GB	gas boiler	$r_{t}$	received reward at time step *t*
ICE	internal combustion engine	$s_{t}$	environment state at time step *t*
ST	storage tank	$V_{i}^{ICE}$	gas consumption of *i*th running ICE, m^3^/h
$a_{t}$	selected action of agent at time step *t*	α,β,γ	electric power consumption coefficients
$c_{t}$	cost at time step *t*, RMB	$\Delta Q_{d}$	difference between supply and demand of cooling power, kW
$C_{total}$	total cost, RMB		
$E^{ST}$	remaining energy storage of ST, kW	$\varepsilon_{LHV}$	low calorific value of natural gas, kWh/m^3^
$n^{AC}$	number of running ACs	$\varepsilon_{peak}$	threshold of peak electric power purchase, kW
$n^{EC}$	number of running ECs	$\eta_{ICE}$	electric efficiency of ICE
$n^{ICE}$	number of running ICEs	θ	extra penalty of reward function
$P_{d}$	electricity load, kW	λ	weighting factors of reward function
$P_{e}$	electric power consumption of supply unit, kW	$\mu_{demand}$	unit price of demand charge, RMB/kW
$P_{e,a}$	allocated electric power consumption of supply unit, kW	$\mu_{gas}$	natural gas price, RMB/m^3^
		$\mu_{grid}$	purchasing electricity price, RMB/kWh
$P_{e,i}^{EC}$	electric power consumption of *i*th running EC, kW	$\mu_{sell}$	selling electricity price, RMB/kWh
		τ	discount factor
$P^{grid}$	exchanging electric power with the grid, kW	ψ	learning factor
$P_{i}^{ICE}$	electric power output of *i*th running ICE, kW	ω/$\omega^{\prime }$	weight parameters of Q network/target network
$P_{rated}^{ICE}$	rated electric power generation, kW		
